# Osmoprotectants play a major role in the *Portulaca oleracea* resistance to high levels of salinity stress—insights from a metabolomics and proteomics integrated approach

**DOI:** 10.3389/fpls.2023.1187803

**Published:** 2023-06-13

**Authors:** Jorge Candido Rodrigues Neto, Fernanda Ferreira Salgado, Ítalo de Oliveira Braga, Thalliton Luiz Carvalho da Silva, Vivianny Nayse Belo Silva, André Pereira Leão, José Antônio de Aquino Ribeiro, Patrícia Verardi Abdelnur, Leonardo Fonseca Valadares, Carlos Antônio Ferreira de Sousa, Manoel Teixeira Souza Júnior

**Affiliations:** ^1^ The Brazilian Agricultural Research Corporation, Embrapa Agroenergy, Brasília, DF, Brazil; ^2^ Graduate Program of Plant Biotechnology, Federal University of Lavras, Lavras, MG, Brazil; ^3^ The Brazilian Agricultural Research Corporation, Embrapa Mid-North, Teresina, PI, Brazil

**Keywords:** purslane, analytical method, chemometrics, metabolomics, proteomics, abiotic stress, salt tolerance, high resolution mass spectrometry

## Abstract

**Introduction:**

Purslane (Portulaca oleracea L.) is a non-conventional food plant used extensively in folk medicine and classified as a multipurpose plant species, serving as a source of features of direct importance to the agricultural and agri-industrial sectors. This species is considered a suitable model to study the mechanisms behind resistance to several abiotic stresses including salinity. The recently achieved technological developments in high-throughput biology opened a new window of opportunity to gain additional insights on purslane resistance to salinity stress—a complex, multigenic, and still not well-understood trait. Only a few reports on single-omics analysis (SOA) of purslane are available, and only one multi-omics integration (MOI) analysis exists so far integrating distinct omics platforms (transcriptomics and metabolomics) to characterize the response of purslane plants to salinity stress.

**Methods:**

The present study is a second step in building a robust database on the morpho-physiological and molecular responses purslane to salinity stress and its subsequent use in attempting to decode the genetics behind its resistance to this abiotic stress. Here, the characterization of the morpho-physiological responses of adult purslane plants to salinity stress and a metabolomics and proteomics integrative approach to study the changes at the molecular level in their leaves and roots is presented.

**Results and discussion:**

Adult plants of the B1 purslane accession lost approximately 50% of the fresh and dry weight (from shoots and roots) whensubmitted to very high salinity stress (2.0 g of NaCl/100 g of the substrate). The resistance to very high levels of salinity stress increases as the purslane plant matures, and most of the absorbed sodium remains in the roots, with only a part (~12%) reaching the shoots. Crystal-like structures, constituted mainly by Na^+^, Cl^−^, and K^+^, were found in the leaf veins and intercellular space near the stoma, indicating that this species has a mechanism of salt exclusion operating on the leaves, which has its role in salt tolerance. The MOI approach showed that 41 metabolites were statistically significant on the leaves and 65 metabolites on the roots of adult purslane plants. The combination of the mummichog algorithm and metabolomics database comparison revealed that the glycine, serine, and threonine, amino sugar and nucleotide sugar, and glycolysis/gluconeogenesis pathways were the most significantly enriched pathways when considering the total number of occurrences in the leaves (with 14, 13, and 13, respectively) and roots (all with eight) of adult plants; and that purslane plants employ the adaptive mechanism of osmoprotection to mitigate the negative effect of very high levels of salinity stress; and that this mechanism is prevalent in the leaves. The multi-omics database built by our group underwent a screen for salt-responsive genes, which are now under further characterization for their potential to promote resistance to salinity stress when heterologously overexpressed in salt-sensitive plants.

## 1 Introduction

The Human Genome Project, completed in April 2003, launched a new era of technological developments in high-throughput biology. Since then, and *vis-à-vis* with an ever-dropping sequencing cost, the world has witnessed the production of many large-scale multi-omics datasets on many different organisms. Datasets in genomics, transcriptomics, proteomics, metabolomics, phenomics, epigenomics, and ionomics, as well as meta-genomics, meta-transcriptomics, and meta-proteomics, are piling up elsewhere, aiming to gain insights on the molecular mechanisms behind several complex living systems. Integrating multiple quantitative molecular measurements with well-designed mathematical models to achieve such a goal is necessary via the combined contribution of many complementary areas of expertise. Multi-omics integration (MOI) uses element-, pathway-, or mathematical-based approaches to integrate omics datasets, opening new windows of opportunities for a further understanding of biological, molecular, and ecological functions and mechanisms; the conceptual integration strategy is sometimes also used (Cavill et al., 2016; Jamil et al., 2020). It is well-accepted that MOI is a non-trivial challenge due to the complexity of most biological systems, some technological limitations, the large number of variables involved, and the necessity for a relatively high number of experimental and biological samples (Santiago-Rodriguez and Hollister, 2021; Vahabi and Michailidis, 2022).

Purslane (*Portulaca oleracea L.*), the most well-known species of the Portulaca genus, is a non-conventional food plant used extensively in folk medicine due to its high nutritional level and wide range of pharmacological effects, involving anti-inflammatory, antibacterial, antioxidant, and anti-ulcerogenic; it is one of the most used medicinal plants, according to the World Health Organization (Zhou et al., 2015). It is multipurpose plant species, serving as a source of features of direct importance to the agricultural and agri-industrial sectors. Multipurpose plants have more than one significant contribution to production and service functions in a land-use system (Srivastava et al., 2021). This species is a suitable model to study the mechanisms of plant tolerance to abiotic stresses not only because of its well-known tolerance to drought and salt stresses but also because of its short life cycle (2–4 months) and the fact that it is a C4 plant that can develop the Crassulacean acid metabolism (CAM) when subjected to water stress and short photoperiod (Koch and Kennedy, 1980; D'andrea et al., 2014; Borsai et al., 2018).

Purslane is an invasive plant and is considered the eighth most common weed in the world (Ozturk et al., 2020). Because of that, its outdoor production in extensive areas faces several concerns. Kong and Zheng (2014) evaluated the potential of producing purslane in a hydroponic system, generating approximately 5.75 kg of fresh matter per m^2^ per month, which might yield 57.5 tons/hectare/year if cultivated in a bimestrial regime. The high productivity of purslane, when grown in controlled-environment agriculture (Vatistas et al., 2022), can open many opportunities for the purslane industry, even in the context of biosaline agriculture (Ozturk et al., 2020).

A robust multi-omics database on the response of purslane to salt stress and its subsequent use via an MOI analysis can create the basis to decode the genetics behind its resistance to salinity stress via a system biology approach (Silva et al., 2022). Most recently, a few reports on single-omics analysis (SOA) of purslane have been published, including Xing et al. (2020), who treated purslane seedlings with 200 mM NaCl and then subjected stems to transcriptome sequencing and metabolome analysis; however, those authors performed SOA but not MOI analysis. Du et al. (2021) produced a high-quality reference transcriptome for purslane, providing a valuable resource for further investigation of related molecular mechanisms, especially the unsaturated fatty acids biosynthesis pathway. Wu et al. (2021) compared the physiological characteristics of two different ecotypes of purslane and analyzed their transcriptome.

The present study is a second step in building a robust database on the morpho-physiological and molecular responses of *Portulaca oleracea L.* to salinity stress and its subsequent use in attempting to decode the genetics behind its resistance to this abiotic stress. After reporting on the characterization of the morpho-physiological responses of young purslane plants to such stress using a robust salinization protocol (Silva et al., 2022), here, we report a study on adult plants through the characterization of the proteome and untargeted metabolome profiles on the leaves and roots of this halophyte species submitted to very high salinity stress and the consequent use of single- and multi-omics analysis strategies to study it.

First, this study aimed to confirm that adult purslane plants presented a higher level of resistance to high salinity stress when compared to young plants. Second, it sought to characterize the metabolome and proteome in the leaves and roots of adult purslane plants. Due to root size in young plants, that was not possible before (Silva et al., 2022). Last, it attempted to integrate metabolome and proteome profiles, aiming to advance in identifying those pathways most affected by this stress in the leaves and the roots.

## 2 Materials and methods

### 2.1 Plant material, growth conditions, experimental design, and saline stress

The B1 accession of purslane (*Portulaca oleracea L.*) used in this study belongs to the Purslane Collection at Embrapa Agroenergia. Seeds underwent disinfection following the same procedure described in Silva et al. (2022), which consisted of soaking in a solution of 2% sodium hypochlorite and Tween® 20 for 5 min, under slow agitation, and subsequent washing with sterile water and drying on sterilized filter paper. After being seeded on a culture medium (MS 1/2 strength, Phytagel 0.2%, and pH 5.8) (Murashige and Skoog, 1962), it was kept for germination in a Growth chamber Conviron mod. Adaptis 1000TC (Controlled Environments, Ltd., Winnipeg, Canada) at 150 μmol/m^2^/s of light and 30°C. After 13 days, seedlings were individually planted in 300-ml plastic cups containing 220 g of sterilized substrate—clay soil, vermiculite, and a commercial substrate (Bioplant®), 2:1:1 (v:v:v) ratio—and transferred to a greenhouse and kept there until the end of the experiments. The plants were allowed to acclimatize for three days, and the salinity stress started 3 weeks after the end of the acclimatization period, exactly 37 days after seeded.

The salinization experiment consisted of two salinity levels (0.0 and 2.0 g of NaCl/100 g of the substrate), with 16 replicates (plants) in a completely randomized design, and the stress lasted 12 days. During the entire experiment, plants were at field capacity. To avoid the loss of Na^+^ or Cl^−^, no leakage of the saline solution was allowed to get out of the plastic cup, as described previously in Silva et al. (2022). The water lost due to evapotranspiration was replaced with deionized water daily, and the electric conductivity at field capacity (ECfc) and water potential in the substrate solution were measured once—on the eighth day of stress—for all replicates (Silva et al., 2022).

### 2.2 Samples for biomass, mineral, metabolomics, and proteomics analysis

Leaves and roots from both treatments—five replicates per treatment—were collected for biomass and mineral analysis. Fifteen samples of substrate were collected for mineral analysis, five before salinization, and 10 at the end of the experiment—five from control and five from stressed plants. After determining the fresh biomass and drying it in a forced air oven at 65°C to constant weight, we measured the dry biomass. Then, we ground it in a Wiley mill Tecnal Mod. TE 680 (Tecnal), passed through a 1-mm (20 mesh) sieve and subjected to extraction of minerals by the standard methods used routinely at Soloquímica (www.soloquimica.com.br). The data from the mineral analysis were submitted to normalization using the Shapiro–Wilk test. For leaves and roots, we applied the t-test, and its non-parametric equivalent was the Mann–Whitney test; and the comparison occurred between two groups (control vs. stressed). For the substrate, the comparison was multiple. At last, ANOVA and its non-parametric Kruskal–Wallis equivalent were performed. Leaves and roots from both treatments—five replicates per treatment—were collected and immediately immersed in liquid nitrogen and then stored at −80°C until extraction of metabolites or proteins.

### 2.3 Metabolomics analysis

Metabolites were extracted using a well-established protocol (Giavalisco et al., 2011; Rodrigues-Neto et al., 2018) that provides polar and lipidic fractions from the same samples. In this protocol, we first ground the plant material (roots or leaves) in a ball mill (Biospec Products, USA) and then added it to a microtube containing 1 ml from a solution (1:3) of methanol and methyl-tert-butyl-ether at −20°C. Samples were incubated for 10 min at 4.0°C and then ultrasonicated for another 10 min in an ice bath. After adding a solution (1:3) of methanol and water to each microtube, they underwent centrifugation (12,000 rpm at 4.0°C for 5 min). The polar (lower) and non-polar (upper) fractions were collected and vacuum-dried in a speed vac system overnight (Centrivap, Labconco, Kansas City, MO, USA). Four microliters of the extract were resuspended in 850 μl of the methanol and water (1:3) solvent mixture and then analyzed by Ultra High Liquid Chromatography coupled to Mass Spectrometry (UHPLC-MS).

For the UHPLC-MS analysis, we used a Nexera X system (Shimadzu Corp., Japan) equipped with an Acquity UPLC BEH C8 reversed-phase column (1.7 μm, 2.1 × 150 mm) (Waters Technologies, USA). The flow rate was set at 400 μl min^−1^, and the column temperature was set at 40°C. The chromatographic runs were isocratic at the start (0–0.5 min) with 4% of B solvent, then at linear gradient (0.5–10 min) with 34% B and (10 – 15 min) with 100% B, and finally isocratic (15–18 min) with 100% B. Solvent A was 0.1% formic acid in water (v/v), and solvent B was 0.1% formic acid in acetonitrile (v/v).

For the mass spectrometry analysis in high resolution, we used a MaXis 4g Q-TOF MS system (Bruker Daltonics, Germany), equipped with an electrospray source both on positive [ESI(+)-MS] and negative [ESI(−)-MS] ion modes. The MS instrument settings used were Endplate offset, 500 V; nebulizer pressure, 4 bar; capillary voltage, 3,800 V; dry gas flow, 9 L min^−1^; and dry temperature, 200°C. The acquisition spectra rate was 3.00 Hz, monitoring a mass range from 70 to 1,200 mass-to-charge ratio (m/z). For external calibration, we used a sodium formate solution (10 mM NaOH solution in 50/50 v/v isopropanol/water containing 0.2% formic acid) directly injected through a six-port valve at the beginning of each chromatographic run. UHPLC-MS data were acquired by the HyStar Application version 3.2 (Bruker Daltonics, Germany).

For data pre-processing, we used the software DataAnalysis version 4.4 (Bruker Daltonics, Germany), where raw data from the UHPLC-MS analysis were exported as.mzXML files. Those files were then submitted to the XCMS Online platform (Tautenhahn et al., 2012; Gowda et al., 2014) for feature detection, retention time correction, and alignment of metabolites detected on each chromatographic run, using parameters optimized based on Albóniga et al. (2020), that creates a more robust data processing through a tuned feature detection to obtain a smaller data matrix with a higher number of SD < 20% variables. We used the centWave for peak detection (maximum peak width = 40 s; minimum peak width = 12 s; Δ m/z = 25 ppm; and mzdiff = 0.002) and minfrac = 0.16, mzwid = 0.02, and bw = 1 used for retention time alignment. Statistics analysis used an unpaired parametric t-test (Welch t-test). Datasets were as follows: eight datasets for leaves samples (control and stressed groups, each with polar and lipidic fraction in both positive and negative modes) and eight datasets for roots samples (following the same logic as the leaves group samples).

After obtaining the pre-processed data from XCMS in.csv files, we used MetaboAnalyst 5.0 for statistical analysis. Data matrices were normalized using an internal standard (ampicillin for polar fraction samples and 1,2-diheptadecanoyl-sn-glycero-3-phosphocholine for lipidic fraction samples). The data structure was kept partially intact, closer to the initial measurements, by employing the Pareto scaling (Van Den Berg et al., 2006). The most popular and well-established statistical tests were performed on the described datasets using MetaboAnalyst were a partial least square (PLS) classification model with its corresponding internal validation (leave-one-out cross-validation method), hierarchical cluster analysis through dendrogram and heatmap using the Euclidean distance measure and Ward clustering algorithm, and a volcano plot to determine up- and downregulated variables using 1.0 as fold change threshold and 0.050 as the false discovery rate (FDR) threshold.

The next step in the statistical protocol was to perform a pathway analysis for leaves and roots, with the collective data of all fractions and ionization modes. The first part of this analysis is through the “functional analysis” module on MetaboAnalyst, done using a mass tolerance of 5 ppm and mixed ion mode, ranked by p-values. The mummichog algorithm in this module was applied using the default p-value cutoff with the latest KEGG (Kyoto Encyclopedia of Genes and Genomes) pathway library version of A. thaliana (Kanehisa, 2000; Kanehisa, 2019; Kanehisa et al., 2021). At the result list, we applied a filter to select the matched compounds with the lowest mass differences in the case of multiple isotopes. Then, the putative annotation was performed on the basis of the exact mass and the Kyoto Encyclopedia of Genes and Genomes (KEGG) compound data for the metabolites of interest. The final list of compounds obtained from the functional analysis was submitted to the pathway analysis using a scatter plot as the visualization method, hypergeometric test as the enrichment method, and relative-betweenness centrality as the topology analysis system.

Acetonitrile Liquid Chromatography - Mass Spectrometry (LC-MS) grade, methanol UHPLC grade, formic acid LC-MS grade, sodium hydroxide ACS grade, and methyl-tert-butyl-ether, acquired from Sigma-Aldrich (Merck, USA), and water from a Milli-Q system (Millipore, USA) were used for the metabolomics analysis.

### 2.4 Proteomics analysis

For total proteins extraction, we used a well-established protocol (Bittencourt et al., 2022; Leão et al., 2022), which consisted of using 5.0 g of ground tissue—with 0.02 g/g of polyvinylpolypyrrolidone (PVP) added to it and mixed with 3.0 ml of buffer (50 mM Tris HCl + 14 mM β-mercaptoethanol, pH 7.5) and 30 µl of protease inhibitor. After gently stirring the suspension on ice for 10 min, it was centrifuged at 10,000 G at 4.0°C for 15 min. Subsequently, 1.0 ml of the supernatant was transferred to 2.0-ml microtubes, mixed with 1.0 ml of 10% TCA (trichloroacetic acid) solution in acetone, kept at −20°C for 2.0 h for protein precipitation, and then centrifuged at 10,000 G at 4.0°C for 15 min. The protein pellet was washed twice with ice-cold 80% acetone, followed by centrifugation under the same conditions described above, and then stored at −80°C until protein quantification (Bradford, 1976) and visualization in an Polyacrylamide gel electrophoresis (PAGE) with sodium dodecyl sulfate (SDS) gel. All samples went to the GenOne company (Rio de Janeiro, RJ, Brazil) for protein preparation and LC-MS/MS analysis, following the same procedure described previously in Bittencourt et al. (2022) and Leão et al. (2022).

A label-free quantitation approach using spectral counting by LC-MS/MS passing the samples through a nano-high performance liquid chromatography (EASY 1000; Thermo Fisher, Waltham, MA, USA) coupled to Orbitrap Q Exactive Plus (Thermo Scientific, Waltham, MA, USA) mass spectrometer was employed for global proteomics analysis. An MS scan spectra ranging from 375 to 2,000 m/z were acquired using a resolution of 70,000 in the Orbitrap. We used the Xcalibur software (version 2.0.7) (Thermo Scientific, Waltham, MA, USA) to obtain the data in biological triplicates.

The MaxQuant software version 2.1.3.0, available at https://maxquant.net/maxquant, was employed to process the raw data (.RAW) for protein identification and abundance, together with the Andromeda algorithm, based on probability (Tyanova et al., 2016a) for the control and salt-stressed treatment, both for leaves and roots. For reference, we used the A. thaliana proteome obtained through the UniProt web platform (Proteome ID: UP000006548), and the cysteine carbamidomethylation and methionine oxidation were the fixed and variable modifications considered, respectively.

As a result, MaxQuant software returns a.txt file named “proteinsgroups” that underwent statistical analysis in Perseus software version 2.0.5.0 (Tyanova et al., 2016b), available at https://maxquant.net//perseus. In it, the lfq intensities for each sample get selected to start the workflow. Initially, it was necessary to carry out a series of filters in the matrix to remove potential contaminants, and then, we identified the samples according to the treatment and transformed the raw values to Log_2_.

With the transformed values, it was necessary to filter the intensities based on valid values, where we defined a minimum occurrence of two replicas present for each group, followed by the replacement of the values absent from the normal distribution. After this step, we added the annotation based on *A. thaliana* and performed the matrix normalization by subtracting the median. At last, this matrix allowed us to visualize the results. All analyses followed the configuration standards already established by Perseus. To generate the volcano plot, we adjusted the FDR to 0.05, and s0 is equal to 0.1.

### 2.5 Functional annotation of proteins and integration of metabolomics and proteomics data

Protein function prediction relies on bioinformatics methods to assign Genetic Ontology (GO) to proteins, specifying their molecular functions (MFs), their involvement in biological processes (BPs), and subcellular locations [cellular components (CCs)] (Törönen et al., 2018). It becomes fundamental to understand how the system operates under normal conditions or stress, whether biotic or abiotic (Merino et al., 2022).

The pannzer2 web software, powered by Sans (Koskinen and Holm, 2012; Koskinen et al., 2015), performs high-performance ontology research, allowing the user a robust prediction (Törönen et al., 2018). In this way, we built different multiFASTA files for each organ and treatment—files containing the proteins present only in the control group, apart from the salt-stressed group and those differentially expressed that belong to both groups—we selected the work species and submitted it to analyses.

The results obtained using MetaboAnalyst and Perseus underwent a metabolic pathway–based integration of proteomics and metabolomics data using the Omics Fusion platform (Brink et al., 2016). The input data used were the IDs of each omics, which include UniProt Accession for proteomics and Kyoto Encyclopedia of Genes and Genomes ID for metabolomics. First, data underwent enrichment through several databases (NCBI, KEGG, and UniProt), and, then, the module “Kyoto Encyclopedia of Genes and Genomes feature distribution” was used to map these omics data in known pathways.

### 2.6 Scanning electron microscopy analysis

Twelve days after the beginning of the salinity stress, purslane leaves were harvested and then left to dry in an oven at 45°C for 72 h. The samples were coated with a 13.4-mm-thick layer of gold using a Quorum Technologies© model Q 150T ES metallizer running the QT GOLD program. Finally, we identified the qualitative composition at specific points in high-resolution images obtained using a scanning electron microscope (SEM) with energy-dispersive spectroscopy (EDS) detectors.

## 3 Results

### 3.1 Visual aspects of purslane plants under salt stress

**Figure 1:**
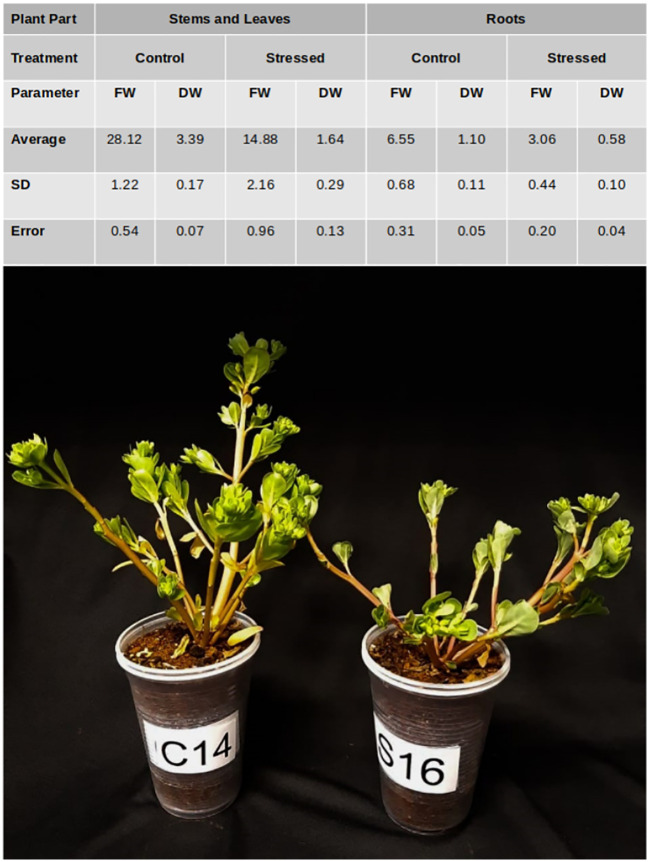
Table with the average fresh (FW) and dry (DW) weight of shoots (stems and leaves) and roots of adult purslane plants grown for 12 days under different concentrations of NaCl (0.0 and 2.0 g of NaCl/100 g of the substrate). Picture of plants at the end of the experiment. C, control; S, stressed; SD, standard deviation.

The water potential in the substrate solution and the electric conductivity at field capacity (ECfc) on the eighth day of stress were −1.3425 MPa and 43.475 dS m^−1^, respectively; values obtained represent an average of 16 replicates (data not shown). The visual aspects of the plant at the end of the experimental period of 12 days were recorded as red, green, and blue (RGB) images. By the end of the experiment, salt-stressed plants showed a general reduction in size and a reduced amount of leaves; meanwhile, no substantial changes were apparent regarding the succulence and color of the stems or leaves. The fresh and dry biomass reduction due to salinity stress was about 50% in the shoots and roots of adult purslane plants; values obtained represent an average of five replicates (**Figure 1**).

### 3.2 Effects of NaCl on the physicochemical properties of the soil mix (substrate) and on the mineral composition of purslane roots and shoots

The salinized substrate used in the cultivation of purslane plants presented higher values for Na^+^, sodium saturation index (SSI), cation exchange capacity, base saturation, and the sum of bases, compared to the control substrate (**Table S1**). In the case of Na^+^, the amount seen in the control was lower than 0.5 mE 100 ml^−1^; meanwhile, the value in the salinized substrate was about 107.5 mE 100 ml^−1^. The SSI in the later substrate was over 40 times higher than in the control treatment.

The mineral composition of the shoots collected from salt-stressed purslane plants at the end of the experiment presented considerably higher amounts of K^+^, S, Cu, and Na^+^ than control plants and a lower Mn. The other macro- and micro-nutrients did not differ much between control and stressed plants (**Table S2**). The roots of salt-stressed purslane plants presented a much higher amount of Na^+^, a slightly higher of N, P, K^+^, and Zn, and a much lower of Mg^+^, Cl^−^, and Fe than the control plants (**Table S2**). No Cl^−^ differences appeared in the shoots, independent of the treatment. However, the amount of this ion in the roots of stressed plants was about 30% of the control. The amount of Na^+^ went from almost zero to 2.3 and 17.4 ppm in the shoots and roots, respectively.

### 3.3 Metabolic fingerprinting analysis

**Figure 2:**
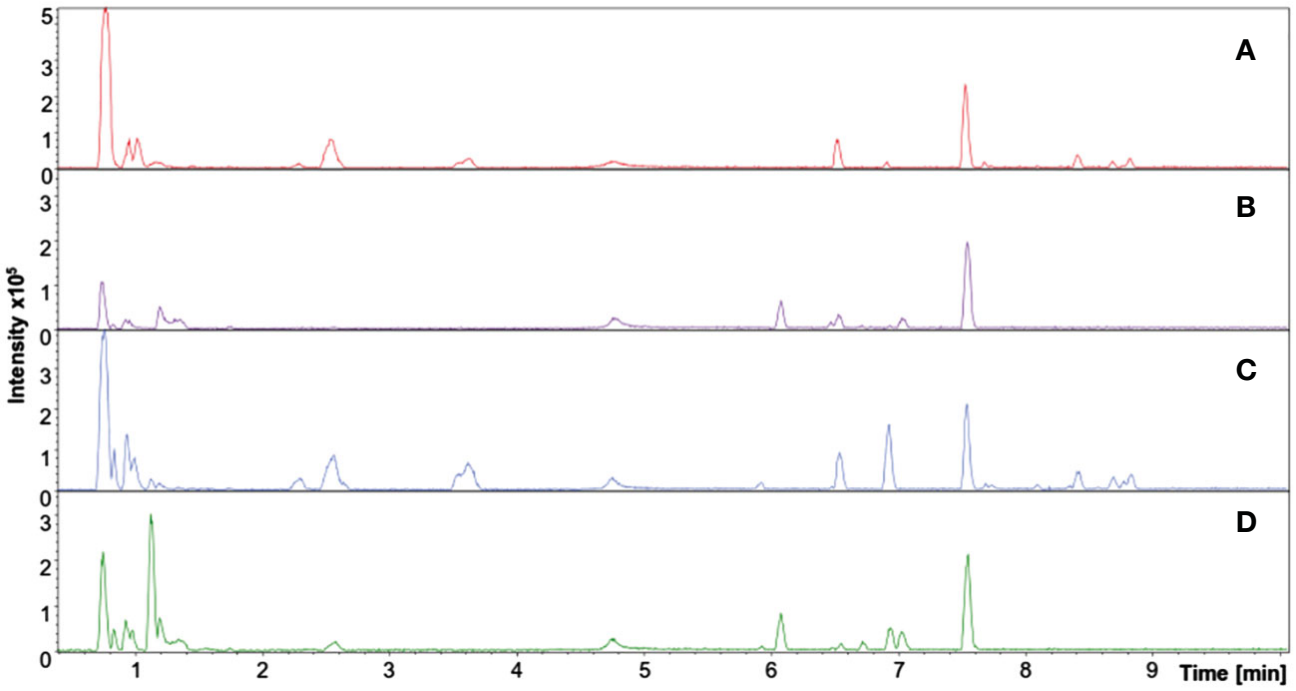
Total ion chromatogram (TIC) of representative samples from adult purslane plants grown for 12 days under different concentrations of NaCl (0.0 and 2.0 g of NaCl/100 g of the substrate). Treatments: **(A)** leaf sample: control group, positive mode ionization; **(B)** root sample: control group, positive mode ionization; **(C)** leaf sample: stressed group, positive mode ionization; and **(D)** root sample: stressed group, positive mode ionization.

Metabolic fingerprinting is an untargeted metabolomics approach based on chromatographic profiles and MS peaks information that might correlate to chemical compounds through annotation. The UHPLC-MS system provides separation on reverse-phase columns and high-resolution mass analysis, providing data able to be statistically processed to give biological information about the control/affected samples. **Figure 2** shows representative chromatographic runs where, through a reverse phase column and a gradient elution, it is possible to infer the segregation of polar compounds at the beginning of the run and less polar compounds at the end of the chromatographic run.

In total, 64 chromatograms resulted from all experimental and analytical conditions—eight for each of the following treatments: roots/control, roots/stressed, leaves/control, and leaves/stressed. Although some peaks were visually different between treatments, most presented few or no perceptible changes in retention time and/or mass intensity. Therefore, a statistical treatment was primordial to avoid interpretation errors.

### 3.4 Metabolomics data analysis

All data from the present study are available via Metabolomics Workbench with project ID PR001633 and DOI 10.21228/M8MM8Q. Data matrices carrying information about masses and intensities from each sample underwent multivariate tests on MetaboAnalyst 5.0, a web-based tool suite for comprehensive metabolomics data analysis that supports several functions for statistical, functional, and data visualization tasks (Pang et al., 2021).

At first, leaves and roots were separately submitted to the one factor statistical analysis module to evaluate group separation between control and stressed samples. Partial least squares-discriminant analysis (PLS-DA), a supervised classification technique that uses regression analysis to rotate components in search of optimal group separation, was used to visualize the effect of salinity on each comparison (Barker and Rayens, 2003). A representative Partial least squares-discriminant analysis score plot grid is presented in **Figure S1**, comparing control and stressed leaves and roots from polar and non-polar fractions on both positive and negative mode ionization. Clear group segregation was observed in all sample fractions and ionization modes through the Partial least squares-discriminant analysis analysis, indicating that the metabolism of leaves and roots changed with the abiotic stress. Cross-validation was employed to ensure the model robustness and classification power through a leave-one-out (LOOCV) model, where all conditions showed acceptable values of Q2 (data not shown).

**Figure 3:**
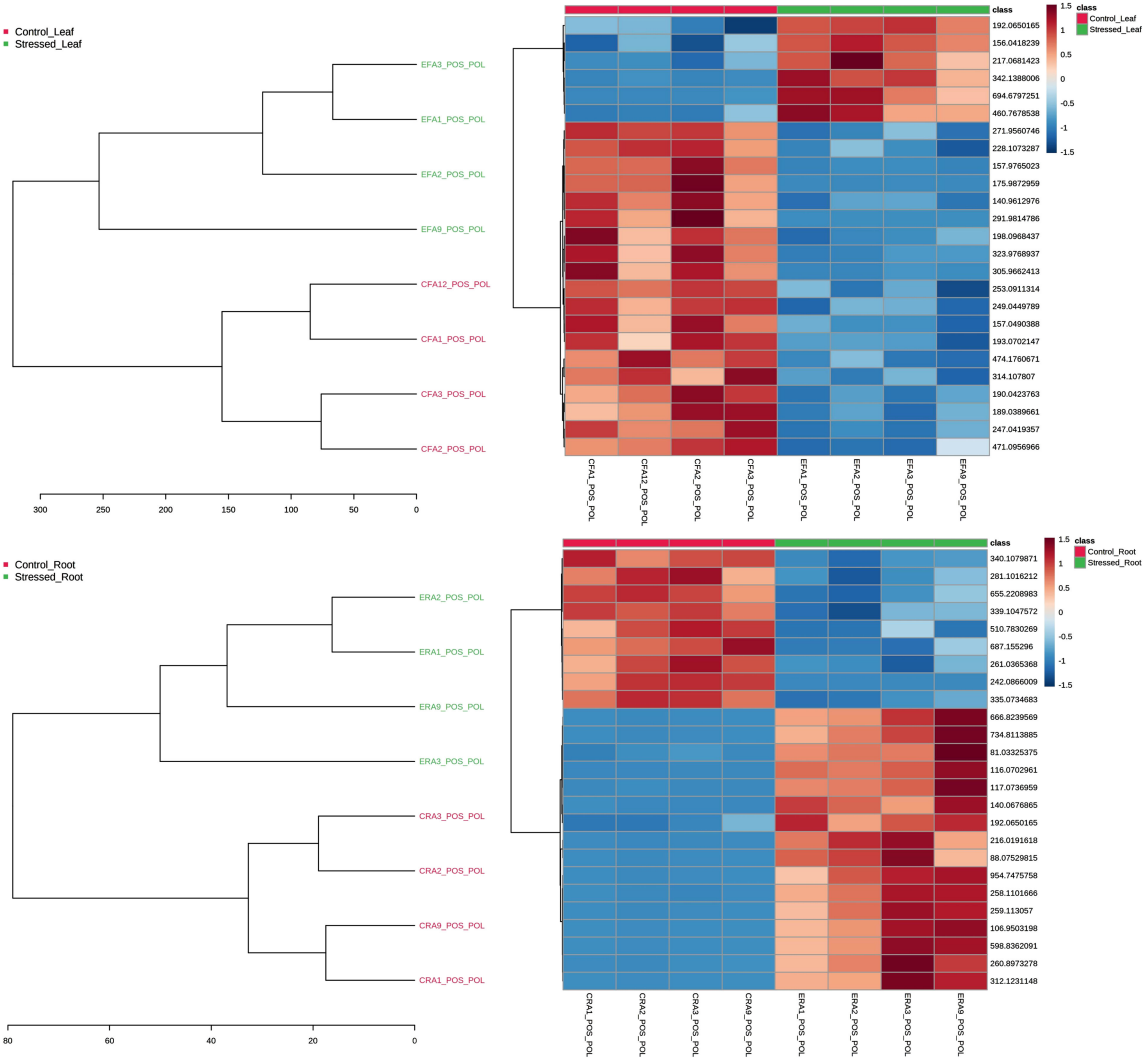
Dendrogram and heatmap used as the hierarchical cluster analysis to evaluate the group and variables separation trends through distance measures. Results from the polar fraction of leaves (top) and roots (bottom) on positive mode ionization. On the heatmaps, the top 25 features, chosen from a previous T-test, are featured.

In the statistical module, a hierarchical clustering analysis (HCA) was also applied to the samples as dendrograms and heatmaps, describing the behavior of variables throughout them using the Euclidean distance measure. One sees the results from the polar fraction of leaves and roots on positive mode ionization in **Figure 3**. The dendrograms plotted as an HCA confirmed the group separation trend with the distances between samples. On the heatmaps, one can observe that among the top 25 features—chosen from a previous T-test—there are features (upper rows) that increase their intensity on stress and those that decrease (**Figure 3**).

**Figure 4:**
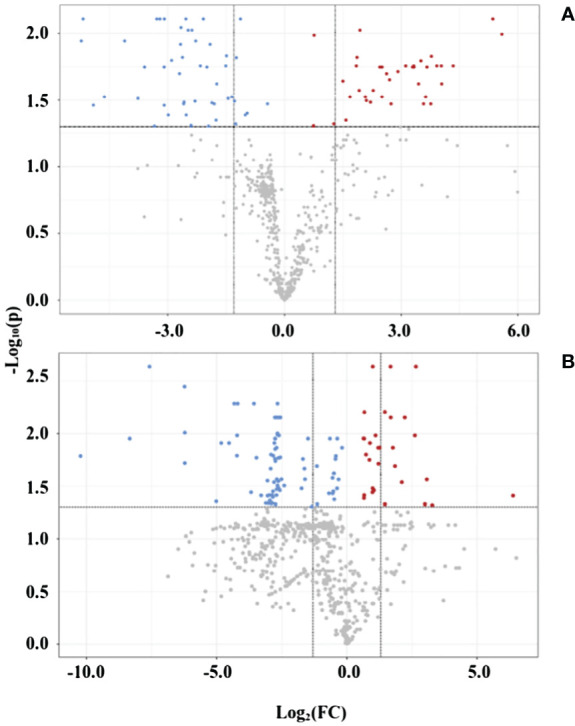
Volcano plot of representative samples from adult purslane plants grown for 12 days under different concentrations of NaCl (0.0 and 2.0 g of NaCl/100 g of the substrate), on polar fraction, positive mode ionization. **(A)** Leaves: blue variables are downregulated (48) and red variables are upregulated (38). **(B)** Roots: blue variables are downregulated (84) and red variables are upregulated (30).

Last, in the statistical analysis module, the volcano plot presents the up- and downregulation of variables regarding specific parameters, such as fold change threshold (set as ≠1, for any change indication) and −log_10_(p) threshold (0.05). As presented in **Figure 4**, the polar fraction in positive mode shows several up- and downregulated peaks used in the upcoming pathway analysis. The fold change of up- or downregulated variables—p-values, FDR, and t-test values—was gathered from all fractions and ionization modes and united in two data matrices: one for leaves and another for roots variables. The mass list of leaves samples had 3,167 peaks, with 306 showing an FDR ≤ 0.05, whereas the mass list of roots had 2,777 peaks, with 206 showing an FDR ≤ 0.05. Those statistically significant variables were organized in a list with their corresponding p-values and ionization modes before undergoing analysis in the functional analysis module.

Leaf and root matrices underwent analysis in the mummichog algorithm, a novel approach to infer metabolites’ putative annotation and pathway activities from a list of mass peaks. The basic premise is that putative annotation at individual compound levels can collectively predict changes at the functional ones, as defined by metabolite sets or pathways. That is because changes at the group level rely on the “collective behavior” that is more tolerant of random errors in the compound annotation. The mass tolerance used for matching m/z features to compounds was 5.0 ppm, and the Kyoto Encyclopedia of Genes and Genomes database used for comparison was from *Arabidopsis thaliana*.

The functional analysis module generated two lists with the differentially expressed peaks and their respective matched compound/adduct, in addition to the corresponding mass difference, for both sample groups (roots and leaves). Multiple isotopes were filtered on the basis of their mass difference and database comparison, as explained earlier (Silva et al., 2022), resulting in the final peak lists used in the pathway analysis. It is worth mentioning that, from the 106 annotated metabolites obtained from the mummichog algorithm on leaves (41) and roots (65) (**Table S3**), there were 13 metabolites differentially expressed in both plant parts: L-tryptophan, glucosamine, betaine, L-arogenate, porphobilinogen, caffeate, (S)-4-amino-5-oxopentanoate, cis-beta-D-glucosyl-2-hydroxycinnamate, nicotinate D-ribonucleoside, 4-hydroxy cinnamyl alcohol 4-D-glucoside, (S)-2-acetolactate, eriodictyol chalcone, and 1-methylxanthine.

The pathway analysis module is the endpoint of the metabolomics study, where metabolites correlate to pathways indicating the biological response of the plant to the applied salinity stress. Information from pathways affected can undergo integrated or complementary multi-omics studies for a broader biotechnological approach. Many techniques have been used in pathway correlation for metabolomics studies, from manual to automated methods (Duarte et al., 2007; Sigurdsson et al., 2010; Jewison et al., 2014; Rodrigues-Neto et al., 2018; Wei et al., 2018). Here, we use the pathway analysis module that integrates methods, such as univariate and over-representation analysis, and novel algorithms and concepts (GlobalTest, GlobalAncova, pathway topology analysis). Both matched compound lists, from the leaves and roots, obtained from the functional analysis, were submitted to the pathway analysis to get a metabolome view graph that contains the arranged p-values (from pathway enrichment analysis) and pathway impact values (from pathway topology analysis).

There were six statistically significant affected pathways in the leaves of adult purslane plants, with a p-value < 0.05, listed here in order of significance (from higher to lower). They were the pentose phosphate pathway; Valine, leucine, and isoleucine biosynthesis; pantothenate and Coenzyme A (CoA) biosynthesis; phenylpropanoid biosynthesis; porphyrin and chlorophyll metabolism; and glycine, serine, and threonine metabolism. On roots, there were also six statistically significant affected pathways, with a p-value < 0.05, listed here in order of significance (from higher to lower): phenylalanine, tyrosine, and tryptophan biosynthesis; phenylpropanoid biosynthesis; indole alkaloid biosynthesis; isoquinoline alkaloid biosynthesis; galactose metabolism; and tyrosine metabolism (**Table 1**).

**Table 1:** List of metabolic pathways in the leaves and roots of adult purslane plants affected by salinity stress.

Plant Organ	Pathway Name	Total	Expected	Hits	Raw p	-log10(p)	Holm adjust	FDR	Impact
**LEAVES**	Pentose phosphate pathway	19	0.487	3	0.011	1.946	1.000	0.610	0.116
	Valine, leucine and isoleucine biosynthesis	22	0.564	3	0.017	1.768	1.000	0.610	0.145
	Pantothenate and CoA biosynthesis	23	0.589	3	0.019	1.714	1.000	0.610	0.093
	Phenylpropanoid biosynthesis	46	1.179	4	0.028	1.558	1.000	0.610	0.084
	Porphyrin and chlorophyll metabolism	48	1.230	4	0.032	1.498	1.000	0.610	0.045
	Glycine, serine and threonine metabolism	33	0.846	3	0.050	1.302	1.000	0.798	0.006
**ROOTS**	Phenylalanine, tyrosine and tryptophan biosynthesis	22	0.949	5	0.002	2.720	0.183	0.135	0.140
	Phenylpropanoid biosynthesis	46	1.985	7	0.003	2.550	0.268	0.135	0.141
	Indole alkaloid biosynthesis	4	0.173	2	0.010	1.983	0.978	0.333	0.000
	Isoquinoline alkaloid biosynthesis	6	0.259	2	0.025	1.609	1.000	0.459	0.500
	Galactose metabolism	27	1.165	4	0.026	1.581	1.000	0.459	0.102
	Tyrosine metabolism	16	0.690	3	0.029	1.542	1.000	0.459	0.007

Only results with a Raw p ≤ 0.05 are shown. Obtained after KEGG IDs of the matched compounds were submitted to the Pathway Analysis module of MetaboAnalyst 5.0, and analyzed using the Hypergeometric Test and the latest KEGG version of the *Arabidopsis thaliana* pathway library.

### 3.4 Proteomics data analysis

All data from the present study are available via ProteomeXchange with the identifier PXD041627 and PXD041628. Approaches to MS-based proteomics analysis are diverse, and deciding which one to use depends on the experimental design and sample preparation (Balotf et al., 2022). The proteomics analysis procedures are label-based or label-free (Anand et al., 2017). The reproduction for protein quantification was determined according to the spectral abundance factors normalized by the MaxQuant and Perseus software in the present study. The raw files obtained through MS are available for consultation upon request for use in other proteomics analysis software.

**Figure 5:**
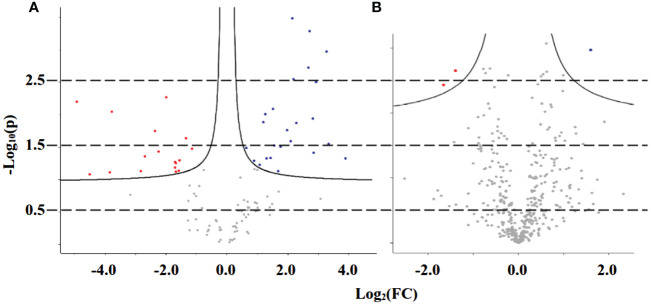
Volcano plot of proteins from adult purslane plants grown for 12 days under different concentrations of NaCl (0.0 and 2.0 g of NaCl/100 g of the substrate), common to both treatments, after analysis that showed statistical significance at the differential expression level, according to abundance. **(A)** Leaves: revealing 42 differentially expressed proteins, 25 of which are positively regulated (blue dots) and 17 are negatively regulated (red dots). **(B)** Roots: revealing only three differentially expressed proteins, one of which is positively regulated and two are negatively regulated.

The analysis of the proteomics profile from purslane leaves led to the identification of 495 proteins, out of a total of 752 entries, that met the specifications, of which 136 proteins came from the control samples and 359 from the stressed ones. After filtering them based on valid values, the proteins were separated into three groups, 103 proteins common to both treatments, 33 present only in control plants, and 256 only in the stressed one (**Figure S2A**). The proteins common to both treatments underwent analysis that showed statistical significance at the differential expression level, according to abundance, revealing 42 differentially expressed proteins (**Table S3**), 25 of which are positively regulated and 17 are negatively regulated (**Figure 5A**).

Nonetheless, the analysis of the proteomics profile from purslane roots led to the identification of 860 proteins that met the specifications, of which 435 proteins came from the control samples and 425 from the stressed ones. After filtering them based on valid values, the proteins did separate into three groups, 397 proteins common to both treatments, 38 present only in control plants, and 28 only in the stressed one (**Figure S2B**). The proteins common to both treatments underwent analysis that showed statistical significance at the differential expression level according to abundance, revealing only three differentially expressed proteins (**Table S4**), one of which is positively regulated and two are negatively regulated (**Figure 5B**).

A set of proteins from the leaves—containing 42 proteins differentially expressed in both treatments, 33 present only in control, and 256 present only in the stressed plants—was then submitted to gene ontology (GO) analysis to classify them accordingly to CC, BP, and MF. Likewise, a set from the roots—containing three proteins differentially expressed in both treatments, 38 only in control, and 28 only in stressed plants—was submitted to GO analysis. Only those groups per GO term with a minimum presence count of three proteins per category are shown in **Supplementary Figures 1**, **2**, except for samples stressed by leaf salt, where a cutoff of 13 or more proteins was employed, simply for better visualization; and for the proteins differentially expressed in the roots, due to the low number.

The BP subgroups with more representatives in the leaves among the proteins found in both treatments (control and stressed plants) were translation and proteolysis (**Figure S3A**). Meanwhile, translation was the only one present in the control plants, and the ones present only in the stressed plants were proteolysis, translation, response to cold, and response to water deprivation (**Figures S3B, C**). The CC and the MF subgroups with more representatives in the leaves, among the proteins present in both treatments, were nucleus and cytosol, and protein binding and mRNA binding, respectively (**Figure S3A**). On the other hand, the CC and the MF subgroups with more proteins present only in the control plants were cytosol and ATP binding, respectively; the same was true in the stressed plants (**Figures S3B, C**).

No BP or MF subgroups had more than one representative among the proteins in both treatments in the roots. In the case of CC subgroups, both mitochondrion and cytosol showed only two proteins among the ones in both treatments each (**Figure S4A**). The CC subgroup with the more representatives in the roots, among the proteins only in the control plants or in the stressed ones, was cytosol. The same was true for ATP binding regarding MF subgroups (**Figures S4B, C**).

At last, those sets of proteins were submitted to the Omics Fusion platform (Brink et al., 2016) for metabolic pathway–based analysis, revealing the metabolic pathways most affected by salinity stress in the leaves and roots of adult purslane plants. In the leaves, the ones with more differentially expressed proteins were Glyoxylate and dicarboxylate metabolism, carbon fixation in photosynthetic organisms, cysteine and methionine metabolism, and glycolysis/gluconeogenesis, all with 12 occurrences. In the roots, cysteine and methionine metabolism and pyruvate metabolism were the pathways with more proteins occurrence, i.e., six (**Table 2**).

**Table 2:** List of metabolic pathways in the leaves and roots of adult purslane plants affected by salinity stress.

Plant Organ	Pathway Name	Pathway ID	Occurrences of Proteins
**LEAVES**	Glyoxylate and dicarboxylate metabolism	630	12
	Carbon fixation in photosynthetic organisms	710	12
	Cysteine and methionine metabolism	270	12
	Glycolysis/Gluconeogenesis	10	12
	Amino sugar and nucleotide sugar metabolism	520	11
	Starch and sucrose metabolism	500	11
	Pyruvate metabolism	620	11
	Glycine, serine and threonine metabolism	260	11
	Carbon fixation pathways in prokaryotes	720	9
	Pentose phosphate pathway	30	9
	Alanine, aspartate and glutamate metabolism	250	9
	Methane metabolism	680	8
	Purine metabolism	230	7
	Arginine biosynthesis	220	7
	Citrate cycle (TCA cycle)	20	7
	Pentose and glucuronate interconversions	40	6
	Arginine and proline metabolism	330	6
	Fructose and mannose metabolism	51	6
	Pyrimidine metabolism	240	5
	Glutathione metabolism	480	5
	Tryptophan metabolism	380	5
	Aminoacyl-tRNA biosynthesis	970	5
	Galactose metabolism	52	5
	Propanoate metabolism	640	4
	Valine, leucine and isoleucine degradation	280	4
	Lysine degradation	310	4
	Vitamin B6 metabolism	750	4
	Fatty acid degradation	71	4
	Porphyrin and chlorophyll metabolism	860	4
	Butanoate metabolism	650	4
	Nitrogen metabolism	910	3
	Oxidative phosphorylation	190	3
	alpha-Linolenic acid metabolism	592	3
	Lysine biosynthesis	300	3
**ROOTS**	Cysteine and methionine metabolism	270	6
	Pyruvate metabolism	620	6
	Amino sugar and nucleotide sugar metabolism	520	4
	Glycine, serine and threonine metabolism	260	4
	Arginine and proline metabolism	330	4
	Pantothenate and CoA biosynthesis	770	4
	Glycolysis/Gluconeogenesis	10	4
	Alanine, aspartate and glutamate metabolism	250	4
	Valine, leucine and isoleucine degradation	280	3
	Carbon fixation in photosynthetic organisms	710	3
	Purine metabolism	230	3
	Arginine biosynthesis	220	3
	Starch and sucrose metabolism	500	3
	Citrate cycle (TCA cycle)	20	3

Only results with three or more proteins occurrence are shown. Obtained using the Omics Fusion platform.

### 3.5 Multi-omics integration analysis

The MOI procedure used to integrate the datasets from the metabolomics and proteomics platforms was a pathway-based mapping using the Omics Fusion platform. Those pathways with ≥ 2 unique molecules differentially expressed are in **Table 3**. The glycine, serine, and threonine metabolism pathway was the most affected when considering the number of proteins and metabolites differentially expressed in the leaves due to salinity stress, with 14 occurrences. Amino sugar and nucleotide sugar metabolism and glycolysis/gluconeogenesis came in second, with 13, and the pentose phosphate pathway in fourth, with 12 (**Table 3**). The following were the most affected pathways in the roots, with eight occurrences each: cysteine and methionine metabolism, purine metabolism, phenylpropanoid biosynthesis, and pyruvate metabolism (**Table 3**).

**Table 3:** List of metabolic pathways in the leaves and roots of adult purslane plants affected by salinity stress.

Plant Organ	Pathway Name	Pathway ID	Occurrences of Proteins	Occurrences of Metabolites	Total of Occurrences
**LEAVES**	Glycine, serine and threonine metabolism	260	11	3	14
	Amino sugar and nucleotide sugar metabolism	520	11	2	13
	Glycolysis/Gluconeogenesis	10	12	1	13
	Pentose phosphate pathway	30	9	3	12
	Carbon fixation pathways in prokaryotes	720	9	1	10
	Methane metabolism	680	8	1	9
	Porphyrin and chlorophyll metabolism	860	4	4	8
	Fructose and mannose metabolism	51	6	2	8
	Aminoacyl-tRNA biosynthesis	970	5	2	7
	Pyrimidine metabolism	240	5	1	6
	Phenylpropanoid biosynthesis	940	2	4	6
	Tryptophan metabolism	380	5	1	6
	Propanoate metabolism	640	4	1	5
	Butanoate metabolism	650	4	1	5
	Phenylalanine, tyrosine and tryptophan biosynthesis	400	2	2	4
	Pantothenate and CoA biosynthesis	770	1	3	4
	Valine, leucine and isoleucine biosynthesis	290	1	3	4
	One carbon pool by folate	670	2	1	3
	Glycosaminoglycan degradation	531	1	2	3
	Ascorbate and aldarate metabolism	53	2	1	3
	Glycosphingolipid biosynthesis - ganglio series	604	1	1	2
	Sphingolipid metabolism	600	1	1	2
	Riboflavin metabolism	740	1	1	2
	C5-Branched dibasic acid metabolism	660	1	1	2
	beta-Alanine metabolism	410	1	1	2
**ROOTS**	Cysteine and methionine metabolism	270	6	2	8
	Purine metabolism	230	3	5	8
	Phenylpropanoid biosynthesis	940	1	7	8
	Pyruvate metabolism	620	6	2	8
	Glycine, serine and threonine metabolism	260	4	3	7
	Pantothenate and CoA biosynthesis	770	4	3	7
	Glycolysis/Gluconeogenesis	10	4	3	7
	Glucosinolate biosynthesis	966	2	4	6
	Amino sugar and nucleotide sugar metabolism	520	4	2	6
	Citrate cycle (TCA cycle)	20	3	3	6
	Methane metabolism	680	1	4	5
	Porphyrin and chlorophyll metabolism	860	1	4	5
	Arginine and proline metabolism	330	4	1	5
	Alanine, aspartate and glutamate metabolism	250	4	1	5
	Carbon fixation pathways in prokaryotes	720	1	3	4
	Valine, leucine and isoleucine degradation	280	3	1	4
	Lysine degradation	310	2	2	4
	Glyoxylate and dicarboxylate metabolism	630	2	2	4
	Carbon fixation in photosynthetic organisms	710	3	1	4
	Lysine biosynthesis	300	2	2	4
	Arginine biosynthesis	220	3	1	4
	Starch and sucrose metabolism	500	3	1	4
	Tryptophan metabolism	380	2	2	4
	Valine, leucine and isoleucine biosynthesis	290	2	2	4
	Pyrimidine metabolism	240	2	1	3
	Pentose phosphate pathway	30	1	2	3
	Aminoacyl-tRNA biosynthesis	970	1	2	3
	Butanoate metabolism	650	1	2	3
	Nicotinate and nicotinamide metabolism	760	1	1	2
	Vitamin B6 metabolism	750	1	1	2
	D-Glutamine and D-glutamate metabolism	471	1	1	2
	Histidine metabolism	340	1	1	2
	Ascorbate and aldarate metabolism	53	1	1	2

Only results with at least one proteins and one metabolite occurrence are shown. Obtained using the Omics Fusion platform.

**Figure 6:**
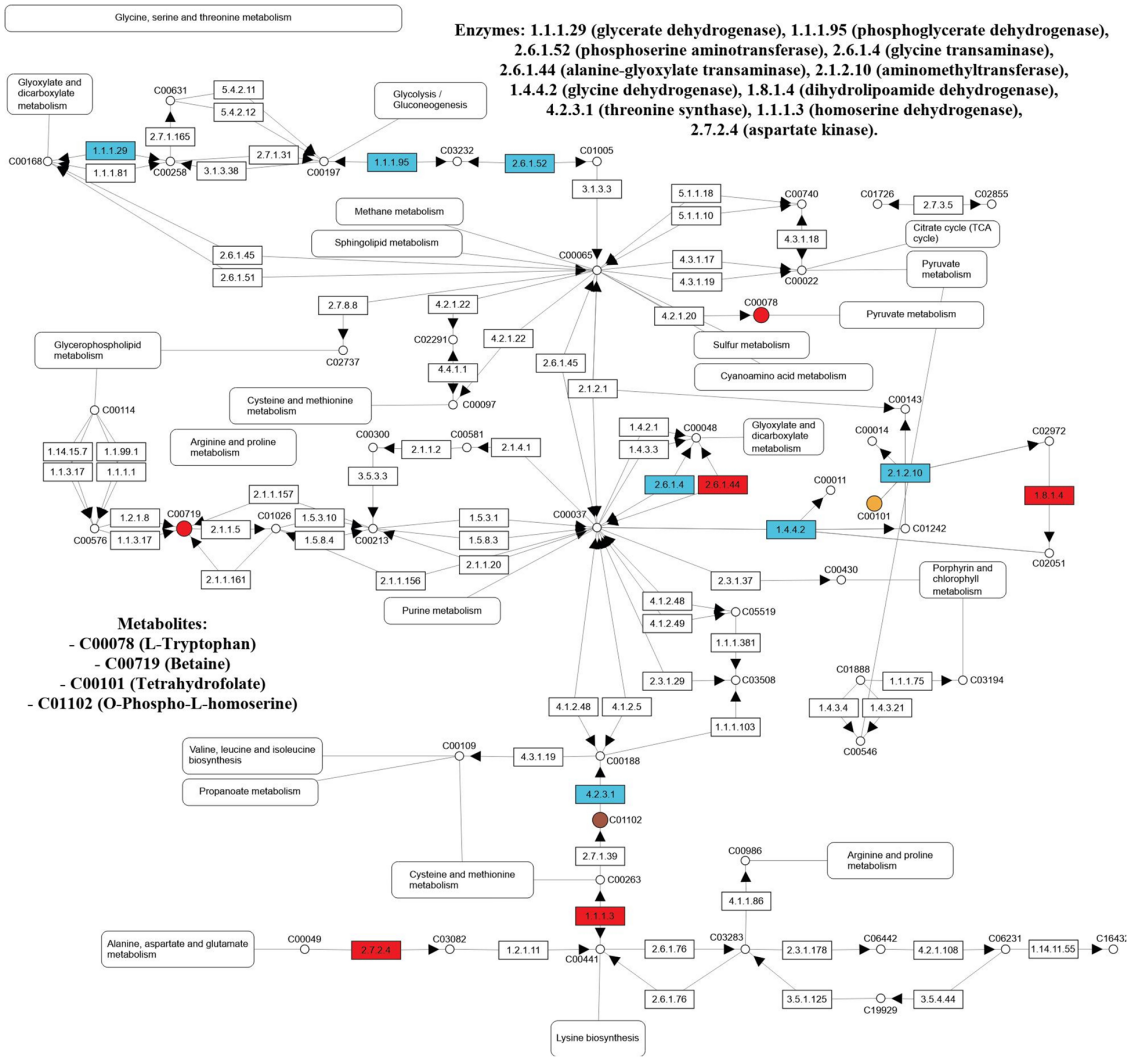
Enzymes (EC number) and metabolites (KEGG Compound number) from the glycine, serine, and threonine (00260) pathway differentially expressed in the leaf and/or roots of adult purslane plants grown for 12 days under different concentrations of NaCl (0.0 and 2.0 g of NaCl/100 g of the substrate). Metabolites differentially expressed are shown as red circles (in both organs), yellow circles (leaves only), brown circles (roots only), and metabolites non-differentially expressed are shown as white circles. Proteins (enzymes) non-differentially expressed are shown as white rectangles, and those differentially expressed are shown as blue (leaves only) and red (in both organs).

In the leaves of salt-stressed adult purslane plants, the glycine, serine, and threonine metabolism pathway had 11 differentially expressed enzymes and three metabolites. The enzymes are glycerate dehydrogenase (EC:1.1.1.29), phosphoglycerate dehydrogenase (EC:1.1.1.95), phosphoserine aminotransferase (EC:2.6.1.52), glycine transaminase (EC:2.6.1.4), alanine-glyoxylate transaminase (EC:2.6.1.44), glycine dehydrogenase (EC:1.4.4.2), aminomethyltransferase (EC:2.1.2.10), dihydrolipoamide dehydrogenase (EC:1.8.1.4), threonine synthase (EC:4.2.3.1), homoserine dehydrogenase (EC:1.1.1.3), and aspartate kinase (EC:2.7.2.4). The metabolites are L-tryptophan (C00078), tetrahydrofolate (C00101), and betaine (C00719) (**Figure 6**).

In other ways, in the roots of salt-stressed adult purslane plants, the glycine, serine, and threonine metabolism pathway had four differentially expressed enzymes and three metabolites. The enzymes are alanine-glyoxylate transaminase (EC:2.6.1.44), dihydrolipoamide dehydrogenase (EC:1.8.1.4), homoserine dehydrogenase (EC:1.1.1.3), and aspartate kinase (EC:2.7.2.4). The metabolites are L-tryptophan (C00078), O-phospho-L-homoserine (C01102), and betaine (C00719) (**Figure 6**).

The enzymes (EC number) and metabolites (Kyoto Encyclopedia of Genes and Genomes compound number) differentially expressed in the amino sugar and nucleotide sugar metabolism and glycolysis/gluconeogenesis pathways, the second most affected in the leaves due to salinity stress, with 13 occurrences of proteins and metabolites each, are presented in **Figure S5**.

### 3.6 Scanning electron microscopy analysis

**Figure 7:**
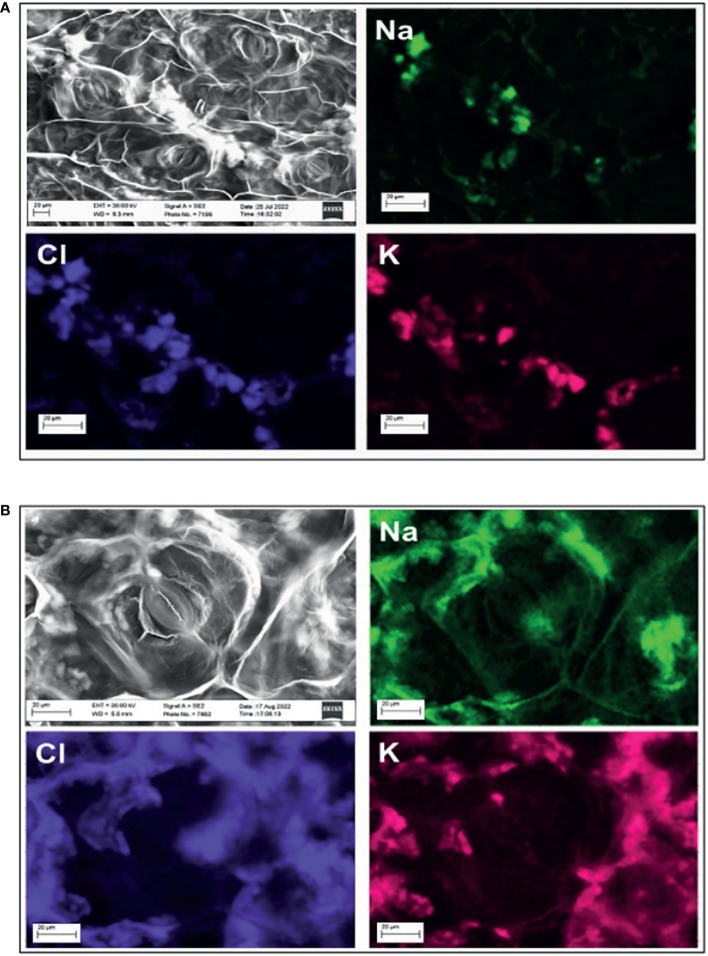
Compositional map of the elements and image obtained by scanning electron microscope (SEM) with detectors of energy-dispersive spectroscopy (EDS) of areas in the leaf of a salt-stressed adult purslane plant. Formation of salt crystals in the leaf vein **(A)** and in the intercellular space **(B)**, near a closed map of the elements showing that the crystals are made of Na^+^, Cl^−^, and K^+^.

In the work of Silva et al. (2022), white crystals surrounding and on the stomata in the leaves of young purslane plants under salinity stress were reported. The compositional map of those crystals showed that Na^+^, Cl^−^, and K^+^ were their constituents, indicating that purslane has a mechanism of salt exclusion operating on the leaves. In the present study, the scanning electron microscopy (SEM) images showed the presence of such crystals in what seems to be the xylem (**Figure 7A**) and intercellular spaces near and around the stomata (**Figure 7B**). Again, the SEM images with detectors of EDS showed that the three above-pointed ions were the main constituents of those crystals (**Figure 7**).

## 4 Discussion

In the present study, adult purslane plants experienced biomass reduction under salinity stress (**Figure 1**). Biomass reduction due to this abiotic stress has been reported before for this species (Borsai et al., 2020; Giménez et al., 2021; Silva et al., 2022). In a previous study done by our group, the salt effects on biomass reduction in young purslane plants were mainly due to the osmotic effect, which results in stomatal closure and restricts the entry of CO_2_ into the leaf mesophyll (Silva et al., 2022). When comparing the response of young and adult purslane plants to a very high level of salinity stress, 40–50 dS m^−1^ (2.0 g of NaCl/100 g of the substrate), it is clear, based on the morphological characterization of the shoots, that the adult plants are less affected by this stress. Consequently, B1 purslane plants become more resistant to salinity stress as it matures. According to Kumar et al. (2021), a decrease in growth and biomass on water dropwort [Oenanthe javanica (Blume) DC] could be due to the adverse effects of salinity on cell division and elongation. In the case of adult purslane plants, reduction in the level of cell division and elongation could also be the reason for the shoots and roots biomass reduction observed in the present study; however, additional studies would be necessary to confirm that.

Adding NaCl to the substrate increased the exchangeable Na^+^ contents by more than a hundred times compared to the control, with a consequent increase in the sum of bases, cation exchange capacity (CEC), and base saturation (BS) (**Table S1**). With more Na^+^ available in the saline treatments, the roots of the purslane plants were able to absorb it in greater quantity (**Table S2**), confirming the premise that salt stress induces the concentration of Na^+^ ions in the plant cells (Borsai et al., 2020; Giménez et al., 2021; Kumar et al., 2021). Much of the absorbed sodium remained in the roots, and a small part was translocated to the aerial part of the plants (**Table S2**), indicating that plants can reduce the damage of salt stress to aboveground tissues by regulating ion transport (Ran et al., 2021; Ran et al., 2022). As for Cl^−^, the roots absorbed only a small part, which translocated to the aerial part of the plants. This result reinforces the assertion that, under salt stress, Na^+^ is the most harmful ion that limits plant growth and causes salt damage (Li et al., 2019).

One of the main consequences of high Na^+^ and Cl^−^ concentrations is the negative effect on the absorption and use of several essential nutrients, causing a nutritional imbalance in the plants (Cruz et al., 2018). Nutrient absorption suffers little or no impact from salt stress in salt-tolerant plants, constituting one of the tolerance mechanisms (Gupta and Huang, 2014; Ran et al., 2021). Maintaining high K^+^ concentration is one of the mechanisms underlying salt tolerance (Britto et al., 2010), and it is what happens in adult B1 purslane plants, which did not change the concentration of other mineral nutrients but experienced an increase in the K^+^ levels in the roots and the shoots of plants stressed by salt. Increasing and maintaining the potassium concentration under saline stress is important, as K^+^ is essential for reducing the cell osmotic potential and keeping the water balance (Kumar et al., 2021; Ran et al., 2021). For Yassin et al. (2019) and Ran et al. (2022), low uptake of Na^+^ and high uptake of K^+^ mean salinity tolerance in higher plants.

The single-metabolomic analysis in the present study identified six pathways in the leaves of adult purslane plants that were affected by the salinity stress (**Table 1**). When comparing the results from adults and young purslane (Silva et al., 2022), considering only those pathways showing a raw p (the original p-value calculated from the enrichment analysis) similar or smaller than 0.05, just two of them appear as affected by the salinity stress in both studies; they are phenylpropanoid biosynthesis and glycine, serine, and threonine metabolism. In young purslane plants, 10 of the 46 metabolites expressed differentially in the phenylpropanoid biosynthesis pathway. That number was only four in the leaves of adult purslane, and only one metabolite expressed differentially in both cases, the cis-beta-D-glucosyl-2-hydroxycinnamate (C05839). On the other hand, in the glycine, serine, and threonine metabolism pathway, six of the 33 metabolites are differentially expressed in the leaves of young plants and three in adult plants. Again, only one metabolite was expressed differentially in both cases: L-tryptophan (C00078).

In the case of single-proteomics analysis, when considering the proteome profile of the leaves from adult purslane plants, the phenylpropanoid biosynthesis pathway is not among those most affected by the salinity stress; differently of what happened in the metabolome profiles of the leaves from young and adult plants. However, the glycine, serine, and threonine metabolism pathway was among the most affected ones in the leaves, having 11 occurrences of differentially expressed proteins (**Table 2**).

Last, the MOI analysis that integrated data from the metabolome and proteome profiles of the leaves from adult purslane plants showed that several pathways were impacted by the salinity stress (**Table 3**). For the sake of this discussion, only the three top ones—accordingly to the number of occurrences—will undergo further consideration, and they are glycine, serine, and threonine metabolism; amino sugar and nucleotide sugar metabolism; and glycolysis/gluconeogenesis.

Considering the results from Silva et al. (2022) and the present study, derived from SOA and MOI studies, it is clear that the glycine, serine, and threonine metabolism is directly involved in the response of purslane plants to salinity stress. That indicates that this pathway plays a role in the resistance to very high salinity stress observed in this halophyte plant species and should be the subject of additional future studies.

Several studies report finding that salinity has a significant impact on glycine, serine, and threonine metabolism. Zhang et al. (2017) showed that this pathway was one of the seven amino acid metabolic ones significantly enriched in the adaption of tomato plants to salt stress. Jia et al. (2020) applied proteomics and metabolomics integration approach to characterize the mechanisms underlying the tolerance of *Malus halliana* Koehne, an apple rootstock, to saline–alkali stress, finding out that this pathway is among the ones most affected by this stress in this species. Derakhshani et al. (2020), using a single-metabolomic analysis approach, reported that salinity significantly impacted this pathway when studying the response of two barley cultivars with contrasting salt tolerance. By comparing the metabolite profiles of three soybean cultivars, Jin et al. (2021) indicated that this pathway and the tricarboxylic acid (TCA) cycle play a role in this plant species’ response to salt stress. Panda et al. (2021) applied a non-targeted metabolomics approach to elucidate the salt tolerance mechanism in *Haloxylon salicornicum*, showing that the glycine, serine, and threonine metabolism was among those pathways playing significant roles in conferring salt tolerance in this xero-halophyte species that grows in saline and arid regions of the world. At last, Wen et al. (2021) reported that the ectopic expression of an MYB30 gene from *Citrus sinensis* in Arabidopsis improves the tolerance to salt stress and drought stress and that this pathway was among those associated with differentially expressed genes between wild-type and the CsMYB30 transgenic plants, showing upregulation of the genes.

Geng et al. (2019) used a single-transcriptomics analysis approach to study sugar beet salt tolerance, reporting that glycine, serine, and threonine metabolism and amino sugar and nucleotide sugar metabolism pathways are among those most significantly enriched in the salt-tolerant cultivar. Using physiological, proteomic, and metabolomic methods to study salt tolerance in pecans, Jiao et al. (2022) showed that amino sugar and nucleotide sugar metabolism was one of the pathways most significantly enriched in pecan plants under salinity stress. By integrating transcriptome and metabolome profiles of mature and plump quinoa seeds imbibed under salinity stress in the dark for 8.0 h, Yan et al. (2022) showed that the amino sugar and nucleotide sugar metabolism significantly enriched. In the present study, this pathway was enriched mainly in the leaves, where all enzymes differentially expressed were regulated positively—present in the leaves of stressed plants but not in the leaves of control plants. On the other hand, in the roots of stressed plants, only one of the four enzymes from the amino sugar and nucleotide sugar metabolism pathway differentially expressed positively regulated under stress. Considering the results from Silva et al. (2022), our studies employing SOA and MOI strategies using transcriptomics, metabolomics, and proteomics data, it is clear that the amino sugar and nucleotide sugar metabolism pathway is also among the most affected by salinity stress but only in adult plants and mainly in their leaves and not their roots. Leaves and roots are plant organs with distinct roles in the response to abiotic stresses, and the role of this pathway in the resistance to very high salinity stress observed in the leaves (but not the roots) of this halophyte plant species should be the subject of additional future studies.

The carbohydrate metabolic process comprises 13 essential pathways, including amino sugar and nucleotide sugar metabolism and glycolysis/gluconeogenesis (Berg et al., 2010). In addition, according to Nong et al. (2019), carbohydrate metabolism is a fundamental metabolic process in living organisms, being one of the most sensitive to salt stress in red dragon fruit, where, within the glycolysis/gluconeogenesis pathway, eight enzymes upregulated after short and continuous saline stress. In studies carried out by Zhang et al. (2019) regarding the mapping of metabolic pathways in sesame subjected to saline stress, the metabolites altered in response to saline stress were mainly involved in amino acid metabolism, oligosaccharide metabolism, citrate cycle (TCA cycle), glycolysis/gluconeogenesis, and urea cycle, suggesting that these metabolic pathways may play roles in the rapid adaptive response to saline stress.

Glucose accumulation suggests downregulation of glycolysis and/or upregulation of gluconeogenesis, which provide metabolic flux for downstream metabolite production to acquire salt tolerance (Llanes et al., 2016). In addition to glucose, the accumulation of other carbohydrates and sugar alcohols, such as fructose, turanose, arabinose, xylitol, D-mannitol, and glycerol, showed significant in *Haloxylan salicornicum*, a xerohalophyte, under salinity, which suggests a role vital role of these metabolites in the regulation of stress-induced oxidative imbalance (Panda et al., 2021). In the present study, beta-D-glucose (C00221) positively regulated in the roots and negatively in the leaves of salt-stressed purslane plants, while alpha-D-glucose 6-phosphate (C00668) negatively regulated in both tissues. At last, the other two metabolites differentially expressed in adult purslane plants under salt stress, beta-D-fructose 1,6-bisphosphate (C05378) and phosphoenolpyruvate (C00074), negatively regulated in the leaves and positively in the roots, respectively.

Osmoprotectants act against the damage caused to the plant’s cellular machinery in response to a stressful environment, protecting the plant (Sharma et al., 2014). They are organic, highly soluble, low molecular weight, electrically neutral, and nontoxic compounds, usually classified into three main groups: a) betaine and associated molecules, b) sugars and polyols, and c) amino acids (Saibi et al., 2020; Zulfiqar et al., 2020; Omari Alzahrani, 2021). Membrane integrity strengthening, enzymatic/antioxidant activity balancing, and water adjustments are physiological responses linked to osmoprotectants’ action in plants submitted to abiotic stresses, including water deficit and salinity (Sharma et al., 2014; Saibi et al., 2020). Different strategies exist to increase salt tolerance in distinct plant species; the exogenous application of osmoprotectants and enzyme engineering are two examples (Omari Alzahrani, 2021). In the present study, betaine was regulated positively in the leaves of adult purslane plants under salinity stress and negatively in the roots. On the other hand, L-tryptophan did upregulate in the leaves and roots of adult plants and leaves of young ones (Silva et al., 2022).

The single-metabolomic analysis in the present study identified six pathways in the roots of adult purslane plants that were affected by the salinity stress (**Table 1**). Silva et al. (2022) did not characterize the metabolomics profile of the roots of young purslane plants because they were too small to be processed, making it impossible to compare the metabolome profiles of the young and adult plants, as done in leaves. However, four pathways affected by the salinity stress in the roots of adult purslane plants also did so in the leaves of young plants. They are phenylpropanoid biosynthesis; phenylalanine, tyrosine, and tryptophan biosynthesis; tyrosine metabolism; and isoquinoline alkaloid biosynthesis. Four of the seven metabolites from phenylpropanoid biosynthesis, differentially expressed in the roots under salinity stress, were also differentially expressed in the leaves of adult purslane plants; they are cis-beta-D-glucosyl-2-hydroxycinnamate (C05839), 4-hydroxycinnamyl aldehyde (C05608), caffeic aldehyde (C10945), and sinapoyl malate (C02887); and those numbers are shorter than the 10 metabolites from this pathway differentially expressed in the leaves of young plants (Silva et al., 2022). Phenylpropanoids are derived from phenylalanine and tyrosine and are involved in plant defense, structural support, and survival. They are phenolics compounds, which are the largest group of secondary metabolites in plants, have antioxidative properties, and can scavenge free radicals, resulting in a reduction of cell membrane peroxidation, hence protecting plant cells from ill effects of oxidative stress (Deng and Lu, 2017; Santos-Sánchez et al., 2019; Sharma et al., 2019). At last, when considering the proteome profile from roots, there was no single pathway common when comparing the list of most affected ones in the metabolome and the proteome.

## 5 Conclusion

After characterizing the morpho-physiological responses of young and adult purslane plants to salinity stress and applying a metabolomics and transcriptomics integrative approach to study the molecular response of young plants and a metabolomics and proteomics integrative approach to do so in adult plants, these are the main conclusions achieved:

a) B1 purslane plants become more resistant to very high levels of salinity stress as it matures.
b) In the case of adult purslane plants, the reduction in cell division and elongation could be the reason for biomass reduction. However, additional studies would be necessary to confirm the following.
c) The roots of the purslane plants were able to absorb Na^+^ in great quantity, and much of the absorbed sodium remained in the roots, with a small part translocated to the shoots.
d) The salt crystal-like structures, previously reported on and around closed stomata on the leaves of young purslane plants (Silva et al., 2022), were also seen in salt-stressed adult purslane plants. As before, we showed that those structures are constituted mainly by Na^+^, Cl^−^, and K^+^ found in what seems to be the phloem and in the intercellular space near the stomata. Once again, it indicates that this species has a mechanism of salt exclusion operating on the leaves, which has its role in salt tolerance.
e) The MOI approach applied reviewed one pathway from the amino acid metabolism [glycine, serine, and threonine (00260)] and two from the carbohydrate metabolism [amino sugar and nucleotide sugar (00520) and glycolysis/gluconeogenesis (00010)] were the most significantly enriched pathways when considering the total number of occurrences in the leaves and roots of adult plants.
f) When considering the enzymes and metabolites expressed differentially under salinity stress within those three pathways, it shows that purslane plants employ the adaptive mechanism of osmoprotection, using various groups of low molecular weight compounds, collectively known as osmoprotectants, to mitigate the negative effect of very high levels of salinity stress and that this mechanism is prevalent in the leaves.

## Data availability statement

The datasets presented in this study will be made public upon publication in online repositories: a) Metabolomics Workbench with study ID ST002537; and b) The mass spectrometry proteomics data have been deposited to the ProteomeXchange Consortium via the PRIDE (Perez-Riverol et al., 2022) partner repository with the dataset identifier PXD041627 and 10.6019/PXD041627, and PXD041628 and 10.6019/PXD041628.

## Author contributions

PA, LV, CS, and MS designed the study. JRN, FS, ÍB, TC, VB, AL, and JAR performed the experiments and generated the data. JRN, FS, and MS wrote first draft of the manuscript, which was extensively edited and approved the submitted version by all authors. MS was responsible for the funding acquisition, project administration, and group supervision. All authors contributed to the article and approved the submitted version.

## Supplementary material

Supplementary Figure 1 Scores plots of representative samples from adult purslane plants grown for 12 days under different concentrations of NaCl (0.0 and 2.0 g of NaCl/100 g of the substrate). Leaves **(A–D)** and roots **(E–H)**. Polar fraction, positive mode **(A, E)**; Polar fraction, negative mode **(B, F)**; Lipidic fraction, positive mode **(C, G)**; and Lipidic fraction, negative mode **(D, H)**.

Supplementary Figure 2 Summary of the proteomics analysis performed on representative samples from adult purslane plants grown for 12 days under different concentrations of NaCl (0.0 and 2.0 g of NaCl/100 g of the substrate). In the leaves, control and stressed conditions shared 103 protein identifications; 33 and 256 proteins were uniquely detected in the control and stressed samples, respectively **(A)**. In the roots, control and stressed conditions shared 397 protein identifications; 38 and 28 proteins were uniquely detected in the control and stressed samples, respectively **(B)**.

Supplementary Figure 3 Gene Ontology (GO) annotation classification statistics graph from proteins in the leaves of adult purslane plants under high salinity stress. Classified accordingly to biological process (BP), cellular component (CC), and molecular function (MF). Numbers represent the amount of positive hits. Proteins present in both treatments (control and stressed) **(A)**, only in the control plants **(B)**, and only in the stressed plants **(C)**.

Supplementary Figure 4 Gene Ontology (GO) annotation classification statistics graph from proteins in the roots of adult purslane plants under high salinity stress. Classified accordingly to biological process (BP), cellular component (CC), and molecular function (MF). Numbers represent the amount of positive hits. Proteins present in both treatments (control and stressed) **(A)**, only in the control plants **(B)**, and only in the stressed plants **(C)**.

Supplementary Figure 5 Enzymes (EC number) and metabolites (Kyoto Encyclopedia of Genes and Genomes Compound number) from the Amino sugar and nucleotide sugar metabolism (00520) and Glycolysis/Gluconeogenesis (00010) pathways differentially expressed in the leaf and/or roots of adult purslane plants grown for 12 days under different concentrations of NaCl (0.0 and 2.0 g of NaCl/100 g of the substrate). Metabolites differentially expressed are shown as red circles (in both organs), yellow circles (leaves only), brown circles (roots only), and metabolites non-differentially expressed are shown as white circles. Proteins (enzymes) non-differentially expressed are shown as white rectangles, and those differentially expressed are shown as blue (leaves only), brown (roots only), and red (in both organs).

Supplementary Table 3 List of differentially expressed peaks (mass to charge ratio—m/z) resulted from the pathway analysis module of MetaboAnalyst 5.0, by organ—leaf or roots. Data set showing the m.z, the matched compound (Kyoto Encyclopedia of Genes and Genomes id), matched form, mass difference, metabolite name, and Log_2_ (FC).

Supplementary Table 4 Proteins from the leaves of adult purslane plants, grown for 12 days under different concentrations of NaCl (0.0 and 2.0 g of NaCl/100 g of the substrate), differentially expressed. Proteins divided in three groups: Present in both treatments, present only in control, and present only in stressed.

Supplementary Table 5 Proteins from the roots of adult purslane plants, grown for 12 days under different concentrations of NaCl (0.0 and 2.0 g of NaCl/100 g of the substrate), differentially expressed. Proteins divided in three groups: Present in both treatments, present only in control, and present only in stressed.
