# Supplementary figures and images for: Osmoprotectants play a major role in the Portulaca oleracea resistance to high levels of salinity stress—insights from a metabolomics and proteomics integrated approach
Source: Front Plant Sci. 2023 Jun 13;14:1187803. doi: 10.3389/fpls.2023.1187803 (PMC10296175; doi:10.3389/fpls.2023.1187803)

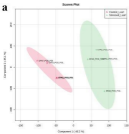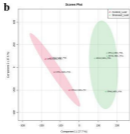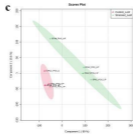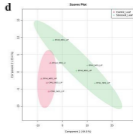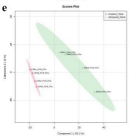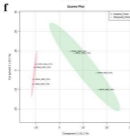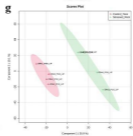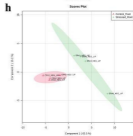

Supplement: Supplementary Figure 1 — Scores plots of representative samples from adult purslane plants grown for 12 days under different concentrations of NaCl (0.0 and 2.0 g of NaCl/100 g of the substrate). Leaves (A–D) and roots (E–H). Polar fraction, positive mode (A, E); Polar fraction, negative mode (B, F); Lipidic fraction, positive mode (C, G); and Lipidic fraction, negative mode (D, H). [file DataSheet_1.zip › Rodrigues Neto et al_Supplementary Material/Figure S1.pdf]

**a**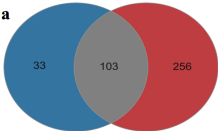

Leaves - Control  
(136)

Leaves - Stress  
(359)

**b**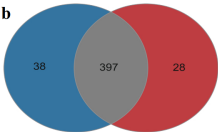

Roots - Control  
(435)

Roots - Stress  
(425)

Supplement: Supplementary Figure 1 — Scores plots of representative samples from adult purslane plants grown for 12 days under different concentrations of NaCl (0.0 and 2.0 g of NaCl/100 g of the substrate). Leaves (A–D) and roots (E–H). Polar fraction, positive mode (A, E); Polar fraction, negative mode (B, F); Lipidic fraction, positive mode (C, G); and Lipidic fraction, negative mode (D, H). [file DataSheet_1.zip › Rodrigues Neto et al_Supplementary Material/Figure S2.pdf]

**a**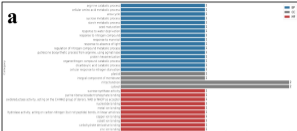**b**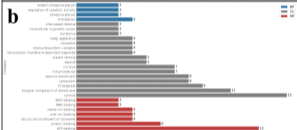**c**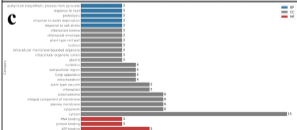

Supplement: Supplementary Figure 1 — Scores plots of representative samples from adult purslane plants grown for 12 days under different concentrations of NaCl (0.0 and 2.0 g of NaCl/100 g of the substrate). Leaves (A–D) and roots (E–H). Polar fraction, positive mode (A, E); Polar fraction, negative mode (B, F); Lipidic fraction, positive mode (C, G); and Lipidic fraction, negative mode (D, H). [file DataSheet_1.zip › Rodrigues Neto et al_Supplementary Material/Figure S4.pdf]

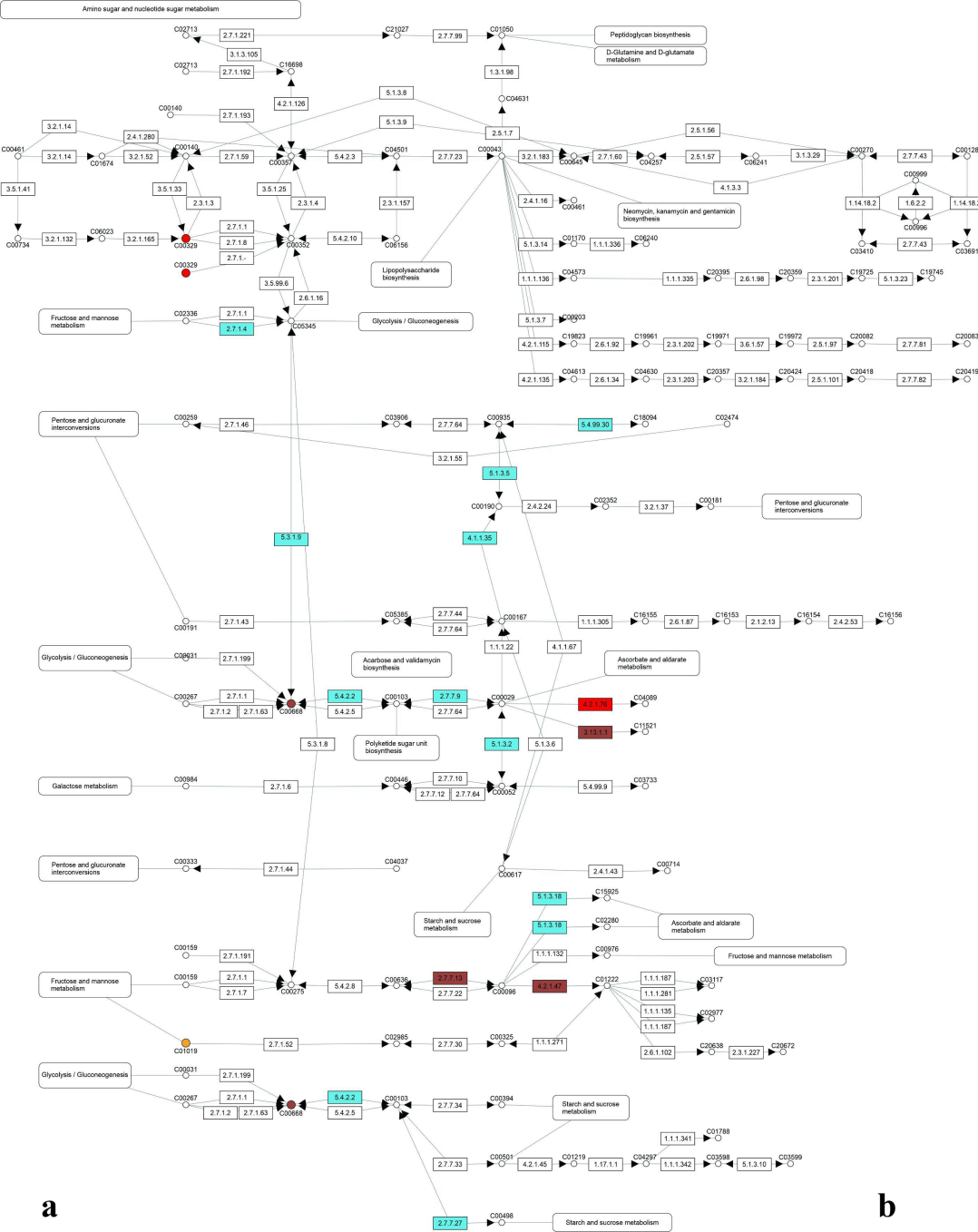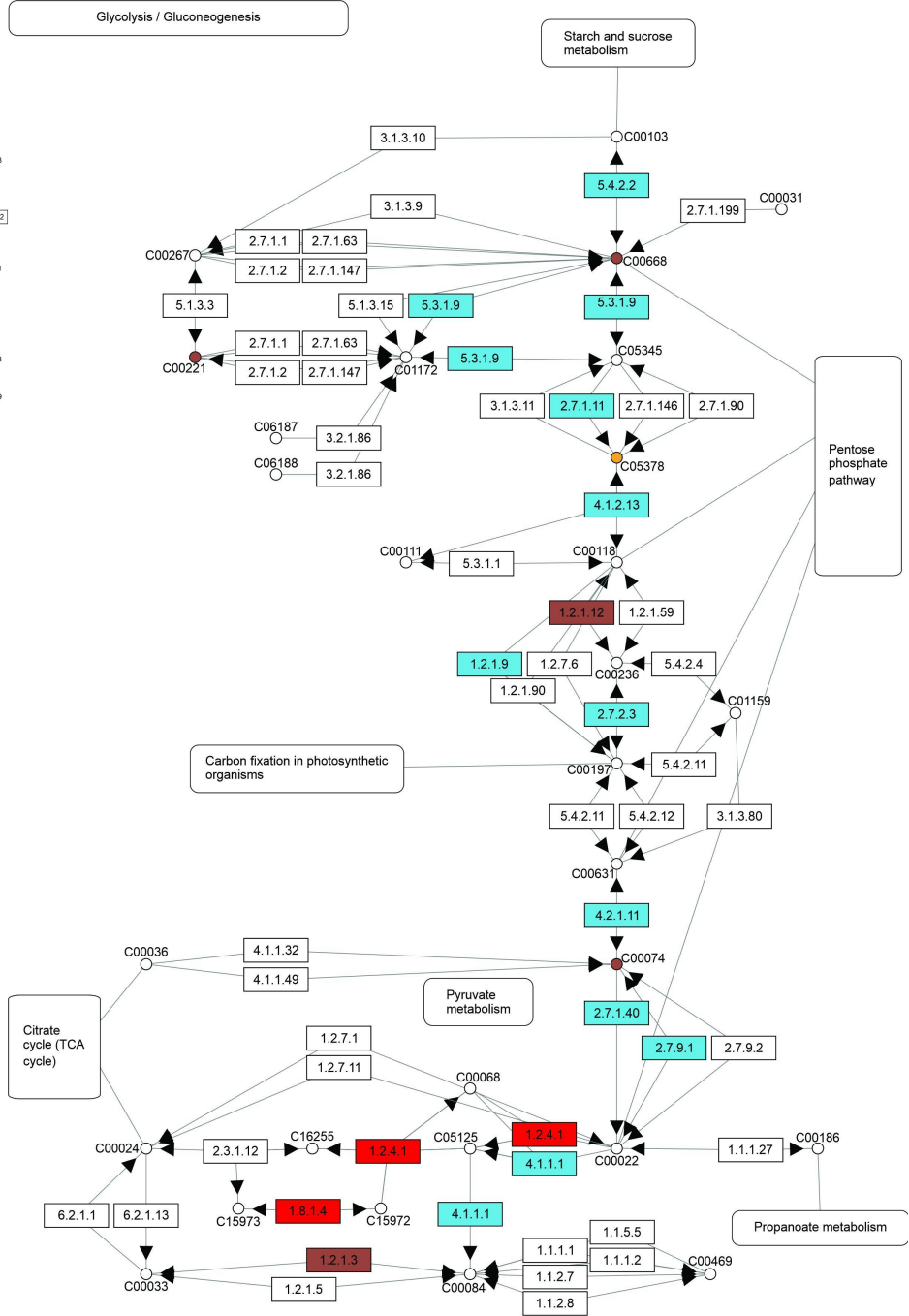

Supplement: Supplementary Figure 1 — Scores plots of representative samples from adult purslane plants grown for 12 days under different concentrations of NaCl (0.0 and 2.0 g of NaCl/100 g of the substrate). Leaves (A–D) and roots (E–H). Polar fraction, positive mode (A, E); Polar fraction, negative mode (B, F); Lipidic fraction, positive mode (C, G); and Lipidic fraction, negative mode (D, H). [file DataSheet_1.zip › Rodrigues Neto et al_Supplementary Material/Figure S5.pdf]
