# Supplementary material for: Osmoprotectants play a major role in the Portulaca oleracea resistance to high levels of salinity stress—insights from a metabolomics and proteomics integrated approach
Source: Front Plant Sci. 2023 Jun 13;14:1187803. doi: 10.3389/fpls.2023.1187803 (PMC10296175; doi:10.3389/fpls.2023.1187803)
Supplement: Supplementary Figure 1 — Scores plots of representative samples from adult purslane plants grown for 12 days under different concentrations of NaCl (0.0 and 2.0 g of NaCl/100 g of the substrate). Leaves (A–D) and roots (E–H). Polar fraction, positive mode (A, E); Polar fraction, negative mode (B, F); Lipidic fraction, positive mode (C, G); and Lipidic fraction, negative mode (D, H). [file Figure S3.pdf › Rodrigues Neto et al_Supplementary Material/Figure S3.pdf]

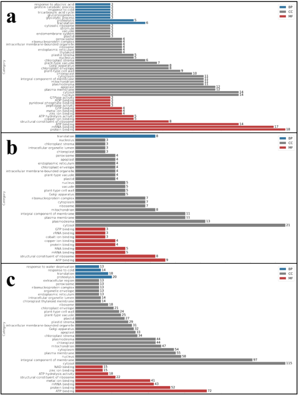
a
BP
CC
MF
Category
response to abscisic acid 3
protein catabolic process 3
response to cold 3
tricarboxylic acid cycle 3
gluconeogenesis 3
glycolytic process 3
proteolysis 5
translation 6
cytosolic ribosome 3
stromule 3
vacuole 3
endomembrane system 3
plastid 3
peroxisome 4
ribonucleoprotein complex 4
intracellular membrane-bounded organelle 4
ribosome 4
endoplasmic reticulum 4
thylakoid 4
plastid stroma 5
nucleolus 5
chloroplast stroma 6
plant-type vacuole 7
Golgi apparatus 8
chloroplast envelope 8
plant-type cell wall 9
chloroplast 10
cytoplasm 11
integral component of membrane 11
mitochondrion 11
plasmodesma 11
apoplast 12
plasma membrane 12
cytosol 14
nucleus 14
GTPase activity 3
ADP binding 3
pyridoxal phosphate binding 3
peptidase activity 3
GTP binding 4
metal ion binding 4
zinc ion binding 4
ATP hydrolysis activity 5
copper ion binding 5
structural constituent of ribosome 8
ATP binding 14
mRNA binding 17
protein binding 18
b
BP
CC
MF
Category
translation 8
nucleolus 3
chloroplast stroma 3
intracellular organelle lumen 3
chloroplast 3
peroxisome 4
apoplast 4
endoplasmic reticulum 4
chloroplast envelope 4
intracellular membrane-bounded organelle 4
plant-type vacuole 4
plastid 4
nucleus 5
vacuole 5
plant-type cell wall 5
Golgi apparatus 5
ribonucleoprotein complex 7
cytoplasm 7
ribosome 7
mitochondrion 8
integral component of membrane 11
plasma membrane 11
plasmodesma 13
cytosol 21
GTP binding 3
rRNA binding 3
cobalt ion binding 3
copper ion binding 4
protein binding 4
RNA binding 5
mRNA binding 5
structural constituent of ribosome 8
ATP binding 9
c
BP
CC
MF
Category
response to water deprivation 13
response to cold 14
translation 18
proteolysis 20
extracellular region 13
peroxisome 13
ribonucleoprotein complex 13
organelle envelope 13
endoplasmic reticulum 13
intracellular organelle lumen 14
chloroplast thylakoid membrane 14
ribosome 18
chloroplast envelope 21
plant-type cell wall 24
plant-type vacuole 25
plastid 27
plastid stroma 29
intracellular membrane-bounded organelle 31
Golgi apparatus 32
apoplast 33
chloroplast stroma 34
plasmodesma 44
chloroplast 44
mitochondrion 47
cytoplasm 54
plasma membrane 55
nucleus 58
integral component of membrane 97
cytosol 115
NAD binding 15
zinc ion binding 15
ATP hydrolysis activity 18
structural constituent of ribosome 22
metal ion binding 41
mRNA binding 43
protein binding 52
ATP binding 72
